# Editorial: How does exercise modify the course of Alzheimer's disease?

**DOI:** 10.3389/fnagi.2023.1127747

**Published:** 2023-01-11

**Authors:** Paul J. Arciero, Patricia Grasso, Cay Anderson-Hanley, Earl Zimmerman

**Affiliations:** ^1^Human Nutrition, Performance, and Metabolism Laboratory, Department of Health and Human Physiological Sciences, Skidmore College, Saratoga Springs, NY, United States; ^2^iPACES LLC, Clifton Park, NY, United States; ^3^Department of Medicine, Albany Medical College, Albany, NY, United States; ^4^Department of Neuroscience and Experimental Therapeutics, Albany Medical College, Albany, NY, United States; ^5^Department of Psychology, Union College, Schenectady, NY, United States; ^6^Department of Neurology, Albany Medical College, Albany, NY, United States

**Keywords:** Alzheimer's disease, cognitive function, leptin mimetics, exerkines, brain-derived neurotropic factor (BDNF), protein pacing

Physical exercise continues to show promise in promoting cognitive health, as well as ameliorating cognitive and physiologic decline. Human and animal studies support various mechanisms of exercise-induced increases in blood flow, exerkines, such as brain-derived neurotropic factor (BDNF) and irisin, and alterations in pro-inflammatory markers, including leptin and interleukin-6 (IL-6), that may contribute to enhanced metabolic and cognitive function. Among the challenges of healthy aging, cognitive decline remains one of the most debilitating and costly. Indeed, Alzheimer's disease and related dementias (ADRD's) continue to plague society with more than 6 million Americans currently diagnosed with Alzheimer's disease (AD) and no cure at present. Likely causes of ADRD's include genetics, lifestyle and environmental factors, whereas the greatest promise for prevention and treatment remains physical exercise. ADRD's exert a heavy toll on health (emotional, physical, and cognitive) and economics of the nation, costing more than $321 billion in 2022 alone, and affecting all segments of the population, particularly marginalized groups such as African American (AA) communities.

This Research Topic explored current evidence-based literature on the impacts of leptin and exerkines on ADRD's, as well as novel insights regarding relationships among peripheral growth factors, vascular function, body composition, exercise training and cognition among AA populations. A universal conclusion from these publications is a consistent pattern of pathologies co-exist, such as diabetes, obesity, and sedentarism, which contributes to the progression of the others, and further exacerbates cognitive decline. Fortunately, physical exercise remains the “miracle drug” to reverse the constellation of these pathologies to preserve cognitive function.

In a comprehensive and well-written review, by Grasso (Harnessing the Power of Leptin), the intricate and complex manner in which leptin manifests its regulatory role in energy metabolism, glucose homeostasis, and cognitive health is discussed. In addition, emerging novel data on synthetic peptide leptin mimetics to mediate leptin resistance associated with metabolic, body composition, and neurologic pathologies is presented. The review details how leptin is involved in cognition and memory *via* receptors in the hippocampus and how plasma leptin concentrations are positively linked with gray matter volume and negatively associated with cognitive decline in elderly humans. Given leptin's ability to serve as both a hormone, mediating numerous endocrine and metabolic pathways, and as a cytokine, augmenting inflammatory responses, its role in ADRD's is more complex. For example, lower plasma leptin concentrations in late-life increases the risk and progression of AD. Whereas, higher leptin increases production of pro-inflammatory cytokines (e.g., TNF-α and IL-6) in the brain, leading to reduced synaptic plasticity and increased progression and symptomatology of ADRD's. Leptin's early promise of combating metabolic and cognitive disease has waned due to its inability to cross the blood brain barrier (BBB) and arrive at its target in the hypothalamus. Grasso states, while leptin administration has not proven efficacious, the burgeoning field of small molecule synthetic peptide leptin mimetics may cross the BBB and specifically target the arcuate nucleus and therefore, does offer promise for metabolic and neurologic health improvement.

Despite numerous risk factors for increased prevalence and progression of ADRD's, cardiometabolic disease and physical inactivity rank among the highest, whereas, physical exercise benefits cognitive, emotional, and physical health, among the elderly. A major neuroprotective effect of exercise relates to prevention of hippocampal volume loss and a concomitant improvement in cognition. Several lines of research support endurance exercise in preserving hippocampal volume in mild cognitively impaired (MCI) patients. The intriguing review by Rody et al., exerkines and Alzheimer's disease, supports physical activity as a proven lifestyle strategy offering neurologic, metabolic, and body composition health protection for ADRD's.

The review emphasizes the role of molecules released during physical exercise, known as exerkines, from either muscle, adipose tissue, liver, or neurons and include; cathepsin B (CTSB), 3-Hydroxybutyrate (3OHB), lactate, IL-6, and BDNF, with a primary focus on irisin, the exerkine released from muscle (referred to as a myokine). Among the many benefits irisin plays in response to exercise training, neuro-modulation likely impacts cognition the most. Impressive work from their lab showed reduced brain levels of irisin in AD patients. In addition, both cognitive performance and BDNF levels were positively associated with irisin and negatively associated with amyloid β levels in human subjects. Rody et al., states plasma irisin is a potent myokine facilitating crosstalk between brain and muscle, which further highlights the therapeutic benefits of exercise on cognition and brain health. The type of exercise that optimizes irisin's communication between muscle and brain warrants further study. Moreover, the authors recommend several methodological strategies to overcome challenges of conducting human clinical trials with individuals with MCI or ADRD's. One suggestion to optimize exerkine release was endurance exercise training of sufficient intensity, duration and frequency and another was implementing exergaming with virtual stimulation to confer both physiological and cognitive benefit.

An additional shortcoming with ADRD experimental designs, is exclusion of African American (AA) populations, which is unfortunate given their increased risk for disease onset and progression. Employing a randomized controlled trial, Gwizdala et al., directly compared a physical activity intervention (PAG) to a successful aging education (SAG) intervention on measures of cognitive function in 56 AA over a 12-week period (African Americans, Exercise and Cognition). The SAG cohort attended weekly 30–60 min group sessions on healthy eating, financial management and dementia awareness, whereas PAG engaged in 150 mins of moderate-vigorous physical activity and two strength sessions per week performed at both home and supervised at a fitness facility. The Repeatable Battery for the Assessment of Neuropsychological Status (RBANS) for cognitive function was administered at baseline and week-12. Surprisingly, only SAG improved cognitive function, with a sub-analysis showing women in SAG improved to a greater extent than men/women in PAG and men in SAG. The authors speculated improvements in SAG compared to PAG may have been due to behavior modifications in multiple domains (e.g., healthy eating, sleep, and financial literacy), and/or group dynamics that facilitated social engagement. Clearly, additional well-controlled clinical trials are needed to interrogate the independent contributions of each intervention.

In an attempt to further tease out meaningful biochemical and physiological factors impacting cognitive function in midlife AA, Traylor et al., analyzed the relationships among BDNF, peripheral vascular function, and body composition with cognition using the CNS Vital Signs computer assessment (BDNF, Vascular Fx, Body Comp, and AA). The investigators employed sophisticated measurement techniques such as Meso Scale Discovery for BDNF quantification, venous occlusion plethysmography for vascular function, and DEXA for body composition. Novel findings showed complex attention and processing speed was associated with BDNF levels and vascular function was related to lean body mass only. These findings support higher blood levels of BDNF in middle-aged AA's is associated with higher functioning brain regions (e.g., prefrontal cortex) required for attention and processing speed. The authors made a strong recommendation to focus future trials on sustaining/building lean muscle mass *via* resistance training for optimal vascular function and BDNF levels for enhanced cognition in mid-life AA's.

Collectively, these published manuscripts contribute meaningful, novel findings regarding specific regulatory mechanisms and lifestyle-related strategies to favorably enhance cognitive health in individuals with mild cognitive impairment and Alzheimer's and related dementias, including the under-represented and higher risk population of AA.

## Author contributions

PA wrote the editorial. All authors read and approved the final editorial.

